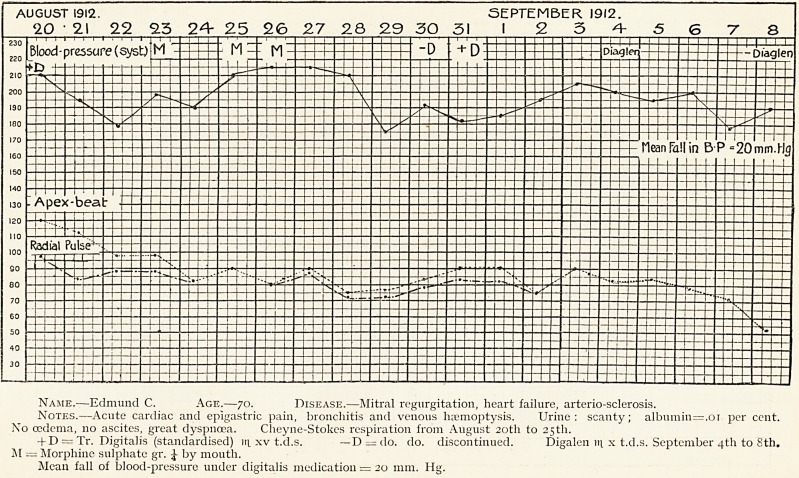# Hyperpiesis and Arterio-Sclerosis

**Published:** 1914-03

**Authors:** C. W. J. Brasher


### HYPERPIESIS AND ARTERIO-SCLEROSIS.

BY

C. W. J. BRASHER, M.D.

---

THE present position of our knowledge of high blood-pressure reveals a want of harmony between the views of the large number of physicians in Europe and America who have directed their attention to this subject.

Much has been written, especially by the American school, which does not find acceptance among the majority of English observers. It may be that the attitude of the latter is too conservative, but they have learned from past experience that the dogmatism of the enthusiast has often retarded true progress.

It is generally admitted that systematic observations of blood-pressure will aid diagnosis and treatment in a number of diseases, but before real progress can be made it would appear to be necessary that two matters of fundamental importance should be settled: (1) The standardisation of the most accurate and reliable instrument or instruments, and (2) the acceptance of a common nomenclature for the various phenomena.

*The Standardisation of Instruments.*—There are at least three types of instruments in common use, mercurial, aneroid and "pressure-gauge."

Although the mercurial instruments are more generally employed, only one of these (French's) is guaranteed to be standardised, and it has been found that the different types often give widely-different readings. It is obvious that careful standardisation of all instruments should be carried out by the makers, and the most reliable type of instrument should be ascertained. It is necessary that the sphygmomanometer should be as accurate as the clinical thermometer, otherwise a large number of observations will be of doubtful value.

In 1913 the writer experimented with several types of aneroid and pressure-gauge instruments, and compared the results with those obtained with French's mercurial sphygmomanometer. He found that the only pressure-gauge instrument giving precisely similar readings at all pressures to those of the mercurial instrument was Hill and Barnard's.

The "Tycos," an American instrument, appeared to be accurate at low and medium pressures, but above 180 mm. there was an error of 20 to 30 mm. Several observers have found that Pachon's sphygmo-oscillometer gives readings about 20 mm. above those of mercurial instruments.[1] Little progress will be made until these errors have been corrected.

Another matter is of even greater importance. Auscultation had been practised in a tentative and uncertain manner by many physicians in the latter part of the eighteenth century. Then came Laennec, who, struggling against ill-health, and in spite of opposition and incredulity of jealous rivals, gave to the world a system of auscultation and a nomenclature that has survived almost unaltered to the twentieth century. As yet no second Laennec has appeared to perform a similar service for sphygmomanometry, and assign its rightful position in scientific medicine.

The absence of any universally-accepted nomenclature has led to much uncertainty, many new terms have been introduced, but they are frequently used in a different sense by various writers. The exact value of pressures other than systolic has not been satisfactorily determined. The majority of English observers consider that the systolic pressure only is of practical value at the present time, but the American writers believe that the diastolic pressure is easily estimated and is of almost equal importance.

As an instance of the laxity with which many terms have been used, some writers appear to think that "high blood-pressure" and "arterio-sclerosis" are synonymous, and if the pulse is described as "excessively hard," and the sphygmomanometer shows a systolic blood-pressure greatly in excess of that which is considered normal for the patient's age and sex, a diagnosis of arterio-sclerosis is made regardless of the condition of the arterial wall. As Herbert French says, "Merely finding a systolic blood-pressure of 150 or 160 mm. Hg. is no proof of granular kidney or of arterio-sclerosis."[2]

It is necessary to remember that in a number of cases a high blood-pressure may exist for a considerable period, probably for years, before definite changes occur in the arterial walls. To this condition Allbutt has applied the term "hyperpiesis."

The recognition of this fact is a point of the utmost importance, as should we be fortunate enough in seeing the patient before definite thickening and degeneration of the arteries have occurred, much may be done to relieve the pressure upon the heart and kidneys, and to restore the patient to comparative if not to perfect health; on the other hand, if this high blood-pressure persist it will result eventually in hypertrophy and dilatation of the left ventricle, followed by chronic interstitial nephritis and the typical changes in the arterial system.

Some have written of arterio-sclerosis as though it were a disease, a distinct pathological entity, and not merely a symptom of some underlying disease which produces many others, such as vertigo, tinnitus, headache and palpitation. The physician's aim should be to ascertain as far as possible the nature of the underlying condition that produces this train of symptoms.

Hyperpiesis is found in many conditions, sometimes in young adults, but there can be little doubt that if the cause be not removed this functional condition may terminate in the definite pathological lesions of arterio-sclerosis.

Probably the most familiar example of hyperpiesis is found in chlorosis. It has been known for many years that a number of chlorotic girls have a systolic blood-pressure considerably higher than normal, and that this produces dilatation of the left side of the heart. Some have supposed that this was due to an actual plethora or "hydræmia," an hypothesis incapable of actual proof; it seems much more probable that it is due to alimentary toxæmia, or as Andrew Clark termed it, "copræmia." This writer emphasised the great value of saline aperients in chlorosis. These patients have frequently very carious septic teeth, pyorrhœa, chronic gastritis and obstinate constipation, with an excess of B. coli in the fæces. Nothing can be more rational than their treatment by saline aperients, which remove large numbers of putrefactive organisms and their products. The result is that the hard, wiry pulse quickly becomes soft and the blood-pressure falls to normal.

It is probable that alimentary toxæmia is the underlying cause of many other cases of hyperpiesis in young or middle-aged adults who are not obviously chlorotic.

Turning now to arterio-sclerosis, the most common causes of this condition are:—

1. Chronic intoxications, alcohol, lead, gout, rheumatism, syphilis.

2. Chronic dyspepsia and constipation, producing alimentary toxæmia, excessive consumption of tobacco, tea, coffee and meat.

3. Sedentary occupations.

4. Prolonged mental or physical strain.

5. Heart disease and chronic nephritis not due to any of the above causes.

Clinical experience shows how frequently are several of these conditions associated. For example, a house painter, aged 48, suffering from chronic plumbism, had excessively thick and tortuous radial arteries. His systolic blood-pressure never fell below 230 mm. Hg. He was a heavy smoker and drinker, and had had syphilis at the age of 20. His teeth were in a most septic state, and he suffered from chronic constipation. The chest was emphysematous, and consequently the cardiac dulness could not be ascertained, but the impulse was very diffuse and "heaving." There was a loud aortic diastolic murmur (probably due to syphilitic aortitis). The urine was of low sp. gr., contained a few casts and a large trace of albumin. In such a case it is difficult to determine the actual cause of the arterio-sclerosis.

Again, many men in middle life, whose occupations are sedentary, eat too much meat, and stimulate their flagging energies with large quantities of alcohol and tobacco. They suffer from chronic constipation, and are often "martyrs to gout."

The frequency of the occurrence of arterio-sclerosis in such individuals is so generally recognised in the United States, that the leading Life Assurance Companies of America require the estimation of the blood-pressure in all cases where the applicant is over 40 years of age.

Arterio-sclerosis occasionally occurs in patients (more frequently women) in early middle life who have never suffered from any serious illness, but who have a "rheumatic" family history, and who have only slight evidence of arthritic changes.

The following cases illustrate this.

**Case 1.**—Miss A. M. H., a school teacher, aged 39, is a life-long total abstainer. Her brother died of cerebral hemorrhage at the age of 38. Two years ago the patient complained of shortness of breath and palpitation. The interphalangeal joints of the fingers have been slightly enlarged for years. The teeth are very good. The pulse was very hard, and the superficial arteries were tortuous and thickened. There was no albuminuria or casts. At the present time she has a systolic blood-pressure of 200 to 210 mm. Hg., and chronic interstitial nephritis.

**Case 2.**—Mrs. E. C., aged 40. Has had three children. She is a very active, energetic woman, who seldom takes any alcohol. Has suffered occasionally from muscular rheumatism, and the interphalangeal joints are slightly enlarged. The mouth is healthy, and she has had very little dyspepsia. No history of constipation. She has severe migraine every few weeks. The urine is normal. She suffers from menorrhagia, and her systolic blood-pressure is seldom below 200 mm. Hg. The radial arteries are very thickened and moderately tortuous.

In addition to "rheumatism," both these patients have had much family trouble, and are of an anxious temperament.

Some families "age prematurely." They are not so well fitted to withstand the stress of modern civilised life as are others of tougher fibre. Many of these persons have prematurely white hair, presbyopia, and other signs of senile degeneration, and although they have avoided errors and excess, arterio-sclerosis often occurs in such cases in early middle life.

*Treatment.*—The causes of high blood-pressure are so diverse and complex, and in many cases have produced such an amount of degeneration of various organs, that the damage is beyond repair, and even our attempts to prevent further arterial degeneration meet with little success. Hence many writers regard endeavours to lower the blood-pressure as likely to result in failure, and they suggest that, did we succeed, the last state of the patient would be worse than the first, because the organs, especially the brain and heart, have been gradually accustomed to working under a greatly augmented blood-pressure, and these authors predict disaster if that pressure be lowered.

The success of treatment of high blood-pressure depends largely upon the condition of the arterial walls, *i.e.* whether the condition is one of hyperpiesis or arterio-sclerosis ; if there be little or no evidence of the latter condition, much may be done in many cases to lower the blood-pressure by elimination of the toxic substances and by regulation of the patient's mode of life, and even in those cases where arterio-sclerosis is present, the patient's life may be prolonged by following Osler's advice, " to open the sluices of the irrigation fields," and " to reduce the speed of the *Mauretania* to that of a ten-knot tramp."

As we have seen, alimentary toxæmia in one form or another plays a most important part in the production of high blood-pressure, and can we but find a suitable intestinal antiseptic, the high blood-pressure when due to septic absorption may be lowered permanently in some cases to a surprising extent.

In an important paper on " Intestinal Disinfection in Alimentary Toxæmia," Frank E. Taylor[3] gives the formulæ of the various amino-acids and toxic amines which have been isolated by Abelous, Dixon and Taylor, and Barger and Walpole. He shows that two of the common products of proteid putrefaction, iso-amylamine and tyramine, are " pressor substances," which possess a markedly similar physiological action to adrenalin.

These investigators have shown that the evolution of a large amount of gas in intestinal putrefaction is due to the decarboxylation of amino-acids, the separation of a molecule of $CO_2$ from the amino-acid reducing it to the corresponding toxic amine.

Hitherto the results of the administration of intestinal antiseptics have been disappointing; camphor, naphthalin, β. naphthol, salol, creasote and phenol, have failed to reduce the number of putrefactive bacteria in the majority of cases in which they have been administered.

Taylor and Hewlett,[4] of King's College, have carried out an important research with "kerol," a coal-tar product which is emulsified with a neutral soap. They state that it has a high carbolic acid coefficient, and is of low toxicity, and that it contains a diphenyl nucleus (thus distinguishing it from phenol). In two normal individuals they found that the number of B. coli in the fæces averaged about ten million per gramme. To one, 10 grains of salol were given thrice daily, and to the other, three, and afterwards six (3 minim) capsules of kerol.

The individual taking salol continued to excrete an undiminished number of B. coli, whilst he who took kerol only excreted 10,000 per gramme, *i.e.* the B. coli content was reduced to $\frac{1}{1000}$ of the former quantity.

"These results were obtained upon healthy individuals eating a normal amount of food. The same results would doubtless be obtained with smaller doses in invalids on smaller and more restricted diets.

"In conclusion, the weight of evidence, both bacteriological and clinical, abundantly proves that the administration of a suitable disinfectant in efficient doses markedly diminishes the production of these substances of bacterial origin, the absorption of which into the blood stream gives rise to the protean manifestations of alimentary toxæmia, and that we now have at hand a ready means of keeping these manifestations in check."

The following case proves this.

Mrs. R. D., aged 83, complained of dimness of sight, vertigo, insomnia, palpitation and frequent "fainting attacks." Patient is thin and fragile-looking. She has never been robust, but has always suffered from dyspepsia and flatulence. She has had no serious illness except "rheumatic gout." Both hands show old typical symmetrical deformities of osteo-arthritis. The patient had lost all her teeth many years ago, and wore a complete artificial set. The mouth was quite healthy. The heart was slightly dilated, and the second sound accentuated. The urine was normal.

When first seen in Oct. 1911, the systolic blood-pressure was 260 mm. Hg., and the pulse rate 96. The patient was kept in bed for several weeks, and prolonged trial of various vasodilators was made, erythrol tetranitrate being the most effectual, but none of these drugs prevented frequent attacks of palpitation and faintness.

On June 21st, 1912, kerol (intestinal) capsules were prescribed, one thrice daily, and in two days the blood-pressure fell from 200 to 140 mm. Hg., and never rose again above 148 mm. The patient made a fairly rapid recovery, and required no further treatment until December, 1913, when she had a return of dyspepsia and flatulence. The blood-pressure before any treatment was commenced was 145 mm. Hg. After the acute symptoms had subsided, kerol was again prescribed, and the patient quickly recovered. Her general health is now better than it has been for years. During the whole time that kerol was being taken there were no toxic symptoms.

This case shows that a very high blood-pressure may exist in an aged patient without producing arterio-sclerosis, that the blood-pressure may be reduced to normal, and that the reduction will persist for eighteen months with great benefit to the patient, and without any of the evil consequences that have been anticipated by some writers.[5]

One other point may be mentioned. Many of these cases of high blood-pressure suffer from symptoms of cardiac failure, with or without valvular disease. Should digitalis be prescribed? Many writers, relying upon the results of laboratory experiments, consider that it should not be given on account of the rapid rise of blood-pressure which it produces in animals when given in large doses. Frederick W. Price [6] has published two papers in the *British Medical Journal* in 1912 and 1913, in which he shows that this rise does not occur in man when digitalis is prescribed in ordinary doses in heart disease. The writer has had several cases of mitral regurgitation, with high blood-pressure, in which the blood-pressure has fallen 20 to 30 mm. during and after the administration of digitalis, and in no case was there any rise in blood-pressure.

This is a point of great clinical importance, but it cannot be discussed adequately here. All that is desired is to suggest that the therapeutics of digitalis require revision from the clinical standpoint, and it may be found that, contrary to accepted views, digitalis or its active principles may be of great service in certain cases of heart failure accompanied by high blood-pressure.

The whole subject of high blood-pressure is so wide that it is impossible to compress it within the limits of a single paper. Charts illustrating several of the cases to which allusion has been made are appended.

REFERENCES.

1 Carey Coombs, *Med. Annual*, 1912, p. 174.
2 *Index of Differential Diagnosis*, p. 18.
3 *Med. Press and Circ.*, 1914, cxlviii. 33.
4 *Ibid.* and *Med. Times*, June 28th, 1908.
5 *Vide* Goodhart, *Practitioner*, 1913, xc. 786.
6 *Brit. M. J.*, 1912, ii. 689 ; 1913, i. 477.

NAME.—Arthur M., tax collector.

AGE.—38.

DISEASE.—Chronic mitral regurgitation, dilatation.

NOTES.—May 13th, 1912: C.O., dyspnœa, palpitation, pain in cardiac region, slight vertigo, Ap.B. diffuse, A.C.D. +, loud rasping apical systolic murmurs. Standardised Tr. Digitalis ♏ xii c.s. t.d.s. from May 13th to 31st. Patient followed usual occupation during treatment, except that he had a holiday for one week (May 24th to 30th). May 31st: All subjective symptoms had disappeared. Ap.B. in normal position, systolic murmur very faint and not conducted outwards. Patient "feels better than he has felt for years."

(X) = Digitalis. (−) = Digitalis stopped.

[*Chart continued on opposite page.*

NAME.—Mrs. R. D. AGE.—83. DISEASE.—Alimentary toxæmia, heart failure, hyperpiesis.

NOTES.—Trinitrin, sodium nitrite, erythrol tetranitrate, all given from January 12th to February 13th. Digalen then prescribed, no vasodilators given subsequently. Blood-pressure taken on each day at 10 a.m. Digalen administration continued until May 30th.

NAME.—Edmund C.  AGE.—70.  DISEASE.—Mitral regurgitation, heart failure, arterio-sclerosis.

NOTES.—Acute cardiac and epigastric pain, bronchitis and venous hæmoptysis. Urine: scanty; albumin = .01 per cent. No œdema, no ascites, great dyspnœa. Cheyne-Stokes respiration from August 20th to 25th.

+D = Tr. Digitalis (standardised) ♏ xv t.d.s.  −D = do. do. discontinued.  Digalen ♏ x t.d.s. September 4th to 8th. M = Morphine sulphate gr. $\frac{1}{4}$ by mouth.

Mean fall of blood-pressure under digitalis medication = 20 mm. Hg.

## Figures and Tables

**Figure f1:**
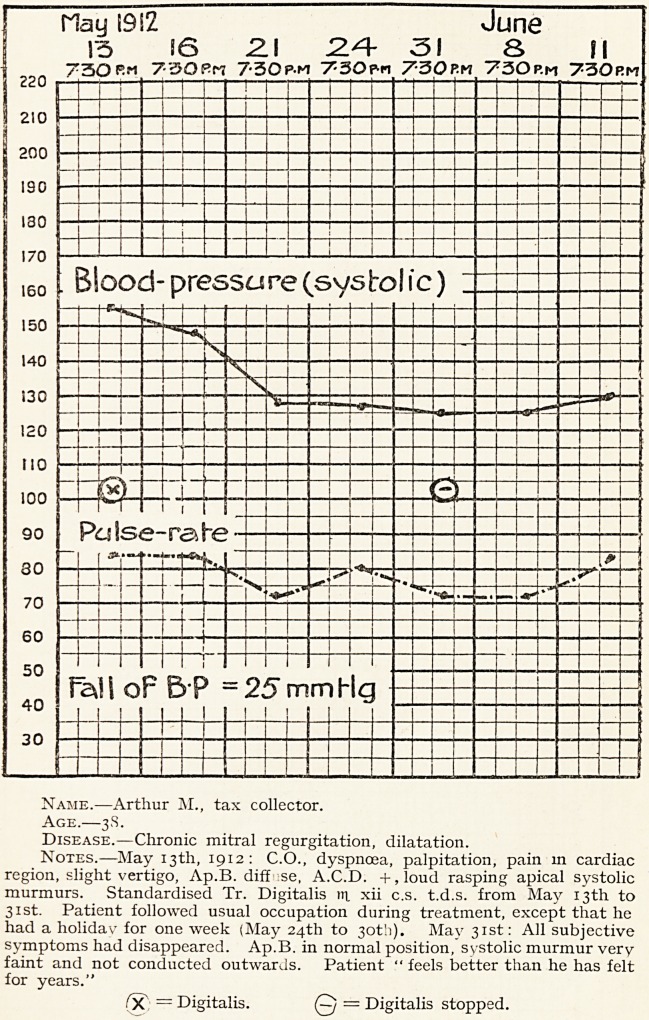


**Figure f2:**
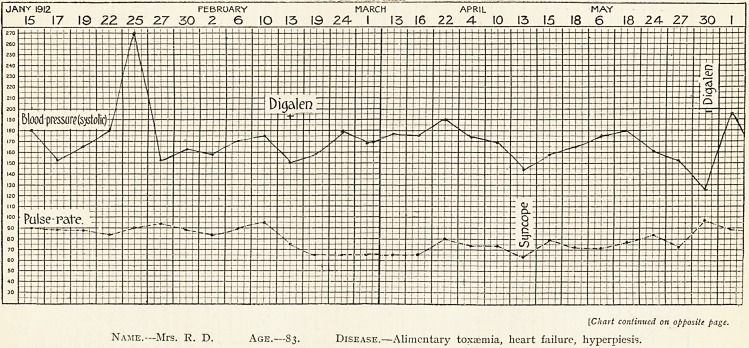


**Figure f3:**
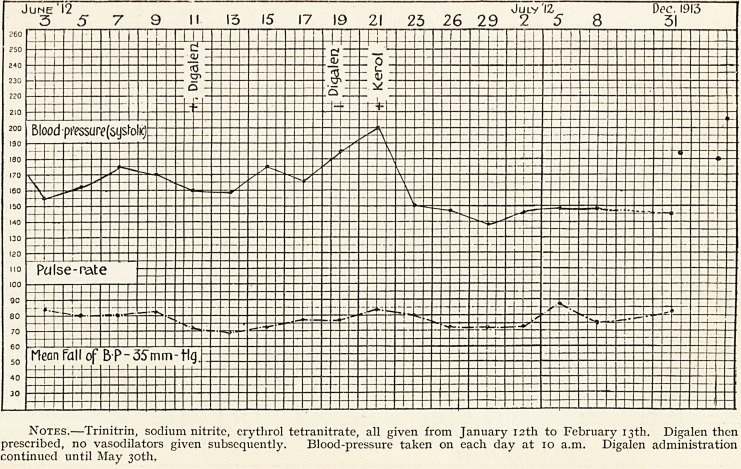


**Figure f4:**